# Progress in Medicine

**Published:** 1898-04-30

**Authors:** 


### Progress in Medicine.

### RHEUMATISM AND GOUT.

(*Continued from page 62.*)

**Treatment.**—Injections of antipyrin have often failed when given for sciatica, but Kuhn[10] considers that this arises from their not being deep enough. He injects a 50 per cent. solution as close as possible to the trunk of the nerve by a long needle, and finds that rapid improvement follows. Salophen seems to be much less toxic than salol and other drugs of the same class, probably because it is only decomposed slowly in the intestine; and Baquet[11] finds that it has none of the drawbacks of the salicylates. He gives four or even six grammes daily in small frequent doses with much success in acute rheumatism and neuralgia, as well as in some skin diseases where there is much itching. Galliard[12] notes its value in acute rheumatism, though even there it is less active than salicylate of soda, but it causes no tinnitus or nausea. Schuller[13] discusses the treatment of chronic villous arthritis, and recommends injections of iodoform 20 parts, pure guiacol 5 parts, and pure acid-free glycerin 250 to 400 parts with every aseptic precaution. He found in the knee that five or six injections were necessary, which caused pain for a day or two, but rarely fever. In more than half the joints treated complete recovery followed. He opened several joints which were badly affected, scraped the diseased membrane and villous growth, sutured them, and then injected his solution, with the result that absolutely normal joints were obtained.

Sokoloff[14] exposed joints affected with acute and chronic rheumatism to the Röntgen rays for ten or twenty minutes at a distance of 50 or 60 centimetres from the tube—a rapid diminution of pain and swelling followed after a few sittings. So much has been said of late as to the value of compresses soaked in oil of winter green to rheumatic joints, that Vida's[15] experiments are interesting. He finds that the natural oil is apt to produce a more or less severe erythema or eczema, while, if the synthetic salicylate of methyl is used, the skin is unaffected. Fifty to a hundred drops may be poured on gauze, covered with oilskin, and wrapped round the limb. This should be renewed every twelve hours.

**Purpura and Allied Diseases.—Henoch's Purpura.**—Dreschfeld[16] enumerates as symptoms pain in or near a joint, a purpuric or erythematous eruption, abdominal pain with vomiting and diarrhœa, and hæmorrhagic nephritis. The stools may contain blood, and the gastric pain is often very severe. The temperature may be normal, or may show an irregular pyrexia, and the spleen is often a little enlarged. Some cases result in chronic Bright's disease, and the diagnosis from rheumatism is at times difficult. Turpentine, quinine, and sulpho-carbolate of soda have been found useful, but the disease is probably due to a bacillary infection. Apert and Rabe[17] report a chronic case of hæmorrhagic purpura which they treated by calcium chloride and citric acid. The symptoms only differed from those of Werlhof's disease by their long duration, and it is noticeable that the blood clot did not contract on standing. Where this non-contractility of the clot exists, the use of calcium chloride is most marked, and in this case it was followed by a rapid cure. Syers,[18] after prolonged observation, fails to trace any relation between rheumatism and purpura as seen in children. In none of his cases could he discover pain or swelling in a joint, but the purpuric rash on the legs quickly disappeared after an improvement of the diet, and was, he thinks, probably a form of scurvy. Singer[19] considers that erythema multiforme is either symptomatic or idiopathic. The latter occurs in spring or autumn and has many relations to rheumatism. The symptomatic type most often follows pyæmic affections; indeed, the writer holds that rheumatism in itself is in a sense a septic blood disease. Examinations of erythema multiforme nearly always show the presence of staphylococci and streptococci. Though many of these eruptions seem to have little connection with rheumatism, a purpuric rash is often observed, as Schonlein pointed out, in many cases of rheumatism. C. T. Aldrich[20] notes an instance where successive attacks were associated with joint symptoms, sore throat and exposure to bad weather, and were promptly relieved by salicylate of soda. On the other hand, nephritis [and abdominal pain like that in Henoch's disease] have been observed in many of these cases, and Osler and others deny that endocarditis follows it.

Another affection allied to rheumatoid arthritis is Quincke's circumscribed œdema. An instance following after an injury to the skull is reported by T. R. Gibson.[21] The patient suffered for some years from gastric crises with patches of œdema on the arms from one to three inches in diameter, which lasted two or three days. There was some tingling but no itching, and the attacks were worse in cold weather. This recurrent affection differs considerably from the circumscribed œdema which sometimes follows poisonous food. H. Oppenheimer[22] reports four cases in which urticaria was accompanied by œdema on the eyelids, feet, hands, and elsewhere. One patient had taken sandal wood, another high venison, a third mussels, and the fourth salicylate of soda for cystitis. The combination of the two rashes, though perhaps not often noted, is, as the writer remarks, not surprising on pathological grounds, for the wheals of urticaria are marked by local œdematous exudations.

**Rheumatoid Arthritis.**—The relations between the true Quincke's œdema and this disease have been pointed out by Jamieson. The question of heart affections in the latter has also been much discussed. They are undoubtedly rare compared to those in rheumatism, but when they do occur, it is doubtful whether they should be considered to be due to a mixed affection or to the organism peculiar to rheumatoid arthritis itself. Bannatyne[23] gives two instances of pericarditis followed by pneumonia, diarrhœa, or pleurisy. Strange to say, the typical joint affections rapidly improved after the attack, though the bacillus found in rheumatoid arthritis was present in them, and the lesions are usually extremely slow to mend. W. Page May[24] has published an account with photographs of bones affected by rheumatoid arthritis from ancient Egyptian cemeteries, which Flinders Petrie announces to be at least 5,500 years old. Sections of the cartilage showed the fibrillation of the matrix, but the evidence of the presence of the Bannatyne-Wohlman bacillus was doubtful. Still, the anatomical proofs of rheumatoid arthritis were complete. The bony outgrowths, the eburnation, and other changes were typical. We may here notice the conclusions of Professor T. Stewart in opening the Montreal debate on this subject.[25] He laid down that the disease is prone to appear in persons of a rheumatic tendency, especially in those suffering from an infectious disease or other depressing agents. There is no proof of the connection with nerve disease or tuberculosis, while recent investigations point strongly to its belonging to the class of infectious diseases. Various speakers drew attention to the analogy between it and gonorrhœal rheumatism or the various types of septic arthritis.

**Gout.**—Luff[26] takes the view that uric acid is formed in the kidneys and absorbed from them into the blood when functional or organic disease exists in them which prevents its excretion from the body. The acid does not exist in normal blood, and is probably formed by the union of urea with glycocine or one of its derivatives. There is no reason to think that deposits occur from a diminished alkalinity of the blood, and the effects of certain wines and beers are due to their action on the liver and the excess of glycocine which it produces in consequence. He advises for treatment citrate of potash, copious draughts of water, hot baths, and the use of a substitute for common salt, which is injurious by hastening the deposit of urates. Even mineral waters which contain chloride of sodium must be avoided. A mixed diet with sufficient vegetables is desirable. In acute attacks he gives first a 30 or 40 minim dose of colchicum, followed by smaller ones with citrate of potash three times a day. A milk diet should be given for a day or two with an initial dose of blue pill and a saline draught. In chronic gout iodide of potash with the tincture of iodine is beneficial if the kidneys are not markedly diseased. Tanage[27] refers to numerous experiments on the power of urotropine to dissolve uratic calculi, and found that the pain and swelling in gout rapidly diminished under its use, and even that tophi were dissolved away. Lartigue's purified extract of colchicum is recommended by the editor of *La Medicine Moderne*. The extract is given in pills covered with a resinous substance that it may pass through the stomach and be dissolved in the intestines. In chronic cases the pills are administered for five days, then left off for ten, and then again given for another week.[28] Haig still adheres to the belief that uric acid exists in the blood, and gave a demonstration of the chloride of ammonium process for its identification.[29] The number of granules shown in a drop of blood by this method corresponded with the amount of uric acid passed in the urine and with the fluctuations caused by the use of certain drugs. He, however, confessed that these granules might be some allied substance, such as xanthin, but insisted that the granules were a measure of the amount of uric acid to be found in the urine of the same individual at the time.

[10] Les Nouveaux Remédes, Mar. 24. [11] B. Med. Jour., Dec. 4. [12] Amer. J. Med. Sc., Dec. [13] Amer. J. Med. Sc., Dec. [14] B. Med. Jour., Dec. 4. [15] Lancet, Jan. 1. [16] Lancet, Dec. 18. [17] Med. Rec., Jan. 29. [18] Lancet, Feb. 1?. [19] B. Med. Jour., Jan. 22. [20] Inter. Med. Mag., Jan. [21] Lancet, Feb. 26. [22] Lancet, Feb. 26. [23] B. Med. Jour., Jan. 15. [24] B. Med. Jour., Dec. 4. [25] B. Med. Jour., Oct. 30. [26] B. Med. Jour., Jan. 15. [27] Therapist, March 15. [28] Med. Rec., Jan. 1. [29] Lancet, March 26.

### DISEASES OF THE HEART AND CIRCULATION.

(*Continued from page* 63.)

**Examination by Rontgen Rays.**—Williams[6] has recorded his experiences in examining the chest by the X rays, based on a very large number of cases. He finds it is better to employ a fluoroscope than to take photographs, as the thoracic organs are in movement, and the movements, whether of respiration or of the heart, are of great importance in making a diagnosis. The trunk appears lighter above than below the diaphragm, and the rise and fall of this muscle are distinctly seen. The chest is divided vertically by an ill-defined dark band, which includes the backbone, on each side of which the lungs, forming the brightest part of the picture, are seen crossed by the darker ribs. The pulsating heart is seen, especially the dark ventricles, the outlines of the venæ cavæ, and of the pulmonary artery, and, under favourable conditions, the lighter right auricle. A small portion of one side of the aorta may be observed in the first intercostal space to the left of the sternum. The best view of the heart is obtained during full inspiration, as then the diaphragm is so depressed as to expose a much greater part than during quiet breathing, and with more air in the lungs the outlines of the heart stand out better on account of the greater contrast thus produced. When the space occupied by the lungs is in a normal condition the outline of the left portion of the heart can be followed by means of the fluoroscope better than by percussion, also the position and size of the right auricle and the right border of the blood vessels, but the right border of the right ventricle, being behind the sternum in health, is better ascertained by percussion. In deep inspiration the heart moves downwards and towards the middle line. The ventricle can be seen to contract and expand in systole and diastole. Changes in position of the heart due to conditions outside of itself—*e g*, effusion, pneumothorax—can be readily seen, or change in position due to its own enlargement. Alterations in size may likewise be detected, *e.g.*, the diminution in size which takes place in anæmia or tuberculosis, or increase from hypertrophy or dilatation. The so-called apex beat, as determined by inspection and palpation, is not the true apex beat in all cases, but sometimes an impulse given by the side of the ventricle. Examination by the fluoroscope also assists in making a differential diagnosis between pericardial effusion and an enlarged heart; the outlines are different, and in effusion the characteristic pulsations of the heart are not seen. This method gives us the means of making an earlier and more certain diagnosis of some cases of thoracic aneurism than any other method, and enables us in certain cases to exclude it where it has been suspected but is not present. It is to be remembered, however, that fluoroscopic examination is only one method, and does not exclude other methods of examination; and its value in assisting to establish a diagnosis varies in different cases.

Thomson[7] gives details of several cases of thoracic disease examined by means of the fluoroscope. One case was that of a patient, aged 49, who complained of paroxysmal attacks of pain in the chest for the past five years. Physical examination showed a systolic aortic murmur, and impairment of percussion, and slight dulness over the manubrium sterni. The vocal cords were normal. Fluoroscopic examination showed that there was some enlargement of the aortic arch of a fusiform character—thus confirming the diagnosis.

Schott[8] has by this means confirmed his previous conclusion, that acute dilatation of the heart can be caused by severe exercise. The heart becomes enlarged in all diameters, but the right ventricle shows the greatest increase, which comes on first and disappears before that of the left ventricle. Thus a bicycle ride of 5½ kilometres (3½ miles) in 23 minutes caused a dilatation of 2 cm. in the right ventricle, and the apex moved outward 1½ cm.

**Pericarditis.**—Shattock[9] has recently reviewed our knowledge of the diagnosis and treatment of pericarditis in a valuable paper. He first says that its chief clinical phenomenon is its latency, *i.e.*, its liability to pass undetected even when especially sought for during life. This is especially the case in pneumonia, where the pulmonary signs mask those of the heart. It also often passes unnoticed in cases of endocarditis. As a general rule, it may be said that the less severe the initial disease the more likely is pericarditis to be detected. Pain is often absent, and the temperature range is of little assistance in framing a differential diagnosis. Friction sounds are the best evidence, but these may pass unnoticed, or be absent. A difficulty in determining the presence of pericardial friction is the occasional existence of a pleuro-pericardial one. If there are no other evidences or suggestions of pleurisy, friction over the pericardium is probably pericardial in origin; but it must be admitted that there are cases where a to-and-fro friction sound isochronous with movements of the heart, and, persisting during arrest of respiration, has turned out to have originated in the pleura contiguous to the pericardium.

Shattock does not believe in the doctrine of a pyriform or pyramidal area of dulness in pericardial effusion; he finds its boundaries identical with those of the enlarged heart, though usually of greater extent. In some very large effusions he has found a loss of resonance below the left clavicle not found on the right side. When the cardiac impulse is perceptible, extension of dulness to some distance to the left of the impulse is an important sign, though it must be admitted that this may be found in dilated hearts. Precordial bulging is rare, and no material assistance in diagnosis is to be found from such occasional symptoms as aphonia, dysphagia, or irritative cough.

[6] Williams, Amer. Jour., Med. Sc., Dec., 1897. [7] Thomson, Lancet, Sep. 16, 1997. [8] Schott, Deutsche Med. Woch., No. 31, 1897. [9] Shattock, Boston Med. and Surg. Jour.

